# Sensitive Measurement of Clinically Relevant Factor VIII Levels in Thrombin Generation Assays Requires Presence of Factor XIa

**DOI:** 10.1055/a-2101-7961

**Published:** 2023-07-10

**Authors:** Tom W. van de Berg, Erik A. M. Beckers, Floor C. J. I. Heubel-Moenen, Yvonne M. C. Henskens, M. Christella L. G. D. Thomassen, Tilman M. Hackeng

**Affiliations:** 1 Department of Biochemistry, Cardiovascular Research Institute Maastricht (CARIM), University Maastricht, Maastricht, The Netherlands; 2 Department of Hematology, Division of Internal Medicine, Maastricht UMC + , Maastricht, The Netherlands; 3 Central Diagnostics Laboratory, Maastricht UMC + , Maastricht, The Netherlands

**Keywords:** thrombin generation, hemophilia, bleeding, FXIa, FVIII

## Abstract

**Background** Hemophilia A (HA) is characterized by decreased or absent factor VIII (FVIII) activity. Current FVIII assays are based on clotting time and thus only provide information about the initiation of coagulation. In contrast, thrombin generation assays (TGAs) can be used to measure the full coagulation spectrum of initiation, propagation, and termination that provide information on the whole course of thrombin generation and inhibition. However, the commercially available TG kits lack sensitivity for measurements of hemophilia plasma within lower FVIII ranges, which is essential for explaining differences in bleeding phenotypes in hemophiliacs at clinically low levels of FVIII.

**Aims** Optimization of the TGA for measurements of low FVIII levels in severe HA patients.

**Methods** TGA measurements were performed in severe HA pooled plasma ( *n* = 10). Investigations of several preanalytical and analytical variables of the assay were performed in a stepwise process and adjusted based on sensitivity toward intrinsic coagulation activation.

**Results** TGA initiated by tissue factor (TF) alone at varying concentrations was unable to significantly differentiate between FVIII levels below 20%. In contrast, TGA activation with low concentrations of TF in presence of FXIa appeared to be highly sensitive for FVIII changes both in high and low ranges. In addition, a representative TGA curve at trough levels could only be produced using the dual TF/FXIa TGA.

**Conclusion** We propose a critical optimization for the setup of the TGA for measurements in severe HA plasma. The dual TF/FXIa TGA shows increased sensitivity, especially in lower FVIII ranges, which allows for better individual characterization at baseline, prediction of interventions, and follow-up.

## Graphical abstract

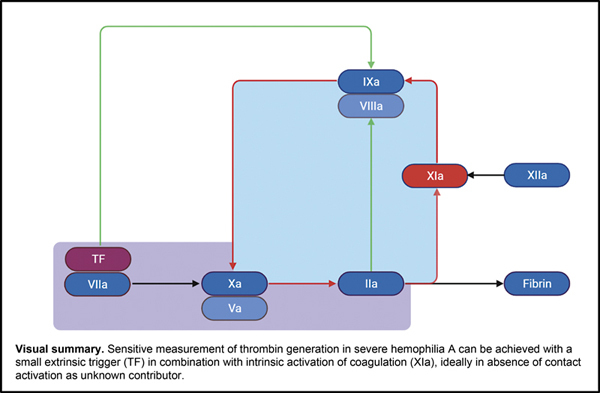

## Introduction

Hemophilia A (HA) is a congenital X-chromosomal bleeding disorder characterized by a deficiency of coagulation factor VIII (FVIII). Its clinical phenotype is classified according to the remaining FVIII activity: less than 1% being severe, 1 to 5% being moderate to severe, and more than 5% regarded as mild. 1 Despite this classification, the severity of the bleeding phenotype within these three groups varies, suggesting that not only the level of FVIII determines the coagulation potential in HA patients.

Global coagulation assays such as the thrombin generation assay (TGA) by calibrated automated thrombography has been mainly used in research settings for the assessment of hypercoagulability. 2 Over the past few years, TGA has seen an increase in applications for hypocoagulability disorders such as HA. 3 4 5 Although TGA offers an attractive option to measure global coagulation potential in HA populations, (pre)analytical standardization is often lacking. Although FVIII measurements are able to precisely measure FVIII levels as low as approximately 1%. By solely focussing on FVIII quantity, both the one-stage and the chromogenic variants are unable to correlate their FVIII measurements with clinical bleeding phenotype. TGA, which is a global coagulation assay, could better reflect the coagulation potential of HA patients as FVIII is measured in its plasma context. However, current TGA assay protocols have a limited sensitivity to low FVIII concentrations. Increasing the assay sensitivity in these lower FVIII ranges, typically at trough levels, might allow for better characterization of individual patients with severe HA and aid in predicting their clinical bleeding tendency.

In this article, we report on optimization of TGA for measurements in low FVIII ranges in HA patients. Based on our results, criteria for optimized TGA are proposed, which might lead to a more precise and accurate estimation of the coagulation potential in individual patients with hemophilia. The optimized hemophilia TGA will aid in analyzing therapeutic effects of different FVIII-based treatments and emerging non-FVIII-based therapeutics. 6 7 8

## Materials and Methods

### Blood Withdrawal and Plasma Preparation

Blood was drawn using vacutainer 21-gauge needles (Becton Dickinson, Plymouth, United Kingdom). Blood was collected in 3.2% citrated vacutainer tubes containing 25 µg/mL thermostable inhibitor of contact activation (TICA) as the contact activation inhibitor. 9 Withdrawal was performed after collection of one small vacutainer clot activator tube, which was discarded. Platelet-poor plasma was made by double centrifugation: whole blood was centrifuged at 2,500 × g for 15 minutes, after which plasma was removed for a secondary centrifugation at 2,500 × g for 15 minutes. Both centrifugation steps were performed at room temperature without centrifugal break. Centrifugation leads to concentration of TICA, resulting in a final concentration of around 50 µg/mL depending on patient sample hematocrit. The resulting plasma was transferred into a new tube and snap-frozen at −180°C and stored at −80°C until use.

### Hemophilia A Plasma Pool

Blood of 10 severe HA patients at trough levels were combined into a hemophilia plasma pool. All patients were on FVIII-replacement therapy. Trough levels were confirmed to be between <1 and 3% with an average of 1.8%. Samples were handled as described above, stored at −80°C and were thawed only once. Patients provided informed consent. The study was conducted in accordance with the ethical standards and principles as agreed upon in the Declaration of Helsinki.

### Thrombin Generation

TGA was performed using calibrated automated thrombography (Thrombinoscope B.V., Maastricht, The Netherlands). 2 TGA was activated by tissue factor (TF; Innovin, Baxter Diagnostics Ltd.) in absence or presence of activated factor XI (FXIa; Innovative Research, Michigan, United States). Recombinant FVIII spiking was performed with octocog alfa (Advate, Takeda Netherlands). The assay was performed in presence of 30 µM phospholipids (PLs) 20/60/20 PS/PC/PE at 37°C. TGA was assessed by addition of the Z-Gly-Gly-Arg 7-amino-4-methylcoumarin low-affinity fluorescent substrate. TF and PLs were incubated for 1 hour in 25 mM Hepes pH 7.4, 150 mM NaCl, 5% BSA (HNBSA 5% buffer). FXIa, when used, was co-incubated with the TF/PL mix for 1 hour. FVIII was incubated with the plasma for 5 minutes before addition of the TF/PL/(FXIa)-mix. Each measurement well consisted of 80 µL plasma, 25 µL CaCl _2_ -containing coagulation trigger (with TF, FXIa, or combinations thereof), and 20 µL of fluorescent substrate. In experiments including FVIII titration, the well setup was altered to 80 µL plasma, 10 µL FVIII in HNBSA 5%, 15 µL coagulation trigger, and 20 µL of fluorescent substrate, totaling 125 µL. The Fluoroskan Ascent Reader (Thermo labsystems, Helsinki, Finland) utilizing a 390/460 nm filter was used in conjunction with the Thrombinoscope software to measure and calculate thrombin generation (TG). All TG measurements included normal pool (NP) samples on the 96-well plates to monitor intra- and inter-assay reproducibility.

## Results

**Fig. 1:**
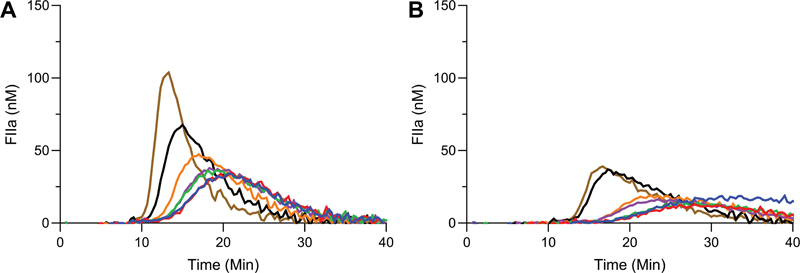
Effect of contact activation on thrombin generation in severe hemophilia plasma. Thrombin generation assays were performed in 2pM TF-initiated severe hemophilia plasma in absence ( **A** ) and presence ( **B** ) of contact activation inhibitor. The plasma was spiked with 0% (blue), 2% (red), 5% (green), 10% (purple), 20% (orange), 50% (black), or 100% (brown) FVIII. Averages of duplicate experiments are shown. FVIII, factor VIII; TF, tissue factor.

The contribution of contact activation during blood drawing and assay procedure was investigated. A FVIII titration in a TF-activated (1 pM) setup was performed in presence and absence of the contact activation inhibitor TICA ( Fig. 1 ) in the blood drawing tube. TF-activated TG in severe HA patient plasma was significantly influenced by contact activation. Peak height and endogenous thrombin potential (ETP) were reduced by 45 to 63% and 23 to 46%, respectively, over the range of added FVIII in presence of a contact activation inhibitor ( Fig. 1 ). As a result, it was decided to add TICA to blood-drawing tubes during collection for our severe HA plasma pool.

**Fig. 2:**
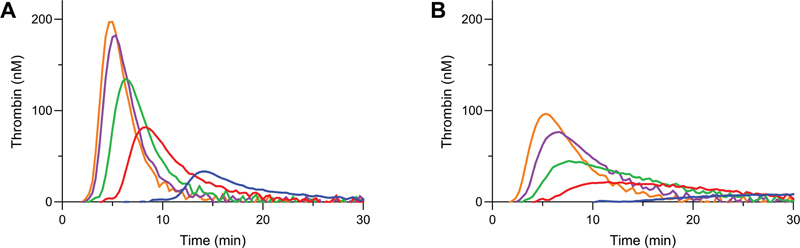
Tissue factor (TF) titration with thrombin generation in normal plasma compared to severe hemophilia plasma. TF titrations (1–5 pM TF) were performed in normal pool plasma ( **A** ) and severe hemophilia A pool plasma ( **B** ). TF was added in concentrations of 1 pM (blue), 2 pM (red), 3 pM (green), 4 pM (purple), and 5 pM (orange). Averages of duplicate experiments are shown.

Next, a TF titration between 1 to 5 pM was performed in NP and HA pool plasma (HAPP). NP plasma showed a gradual increase of both ETP and peak height with increasing concentrations of TF ( Fig. 2A ). All curves in NP plasma showed a representative curve shape reflecting initiation, propagation, and inhibition of TG. TF titration in HAPP, as expected, showed a significantly decreased ETP and peak height compared to NP plasma ( Fig. 2B ). Moreover, TG at both 1 pM and 2 pM TF showed aberrant curve shapes due to lack of a clear peak and due to prolonged tailing. As overstimulation of the extrinsic pathway by an excess of TF might reduce the sensitivity toward the FXI–FVIII propagation loop, the lowest TF concentration with a representative TG curve (3 pM TF) was chosen for further experiments ( Fig. 2B ).

**Fig. 3:**
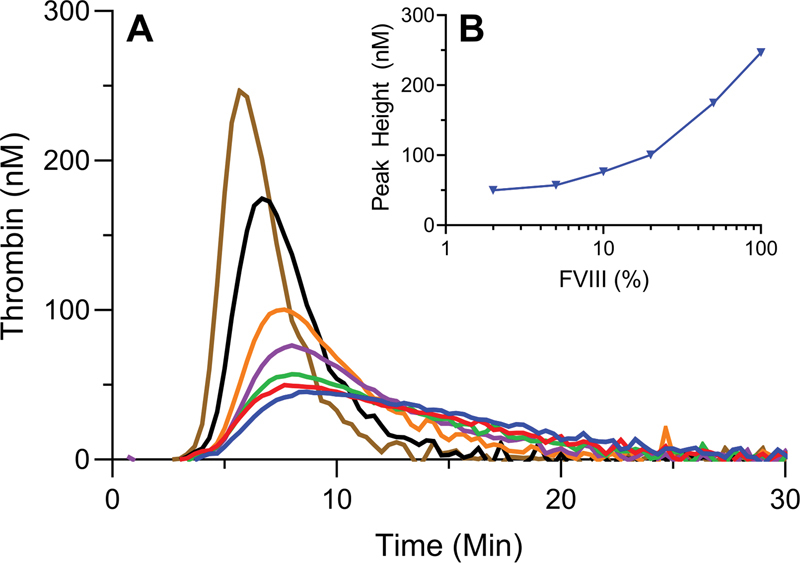
FVIII titration in severe hemophilia plasma activated by 3 pM of tissue factor. Thrombin generation in pooled severe hemophilia plasma was initiated by 3 pM of tissue factor. FVIII titrations of 0% (blue), 2% (red), 5% (green), 10% (purple), 20% (orange), 50% (black), and 100% (brown) were performed ( **A** ). Panel ( **B** ) shows a logarithmic extrapolation of peak heights from the data from panel (A). Averages of duplicate experiments are shown. FVIII, factor VIII.

A FVIII titration TGA was performed in pooled hemophilia plasma, initiated by 3 pM of TF ( Fig. 3A ). FVIII activities between 0 and 5% failed to show a dose-dependent increase of ETP or peak height. Moreover, the shallow curve shape with a flattened peak and prolonged tail did not allow accurate analysis of ETP. In contrast, measurements of TGA in pooled hemophilia plasma with added FVIII levels between 10 and 100% did show a dose-dependent increase of TG in both ETP and peak height with adequate curve shapes. Thus, the sensitivity towards added FVIII levels became apparent only at levels of FVIII of >10% and was less pronounced in the lower, more clinically relevant FVIII ranges of <10% ( Fig. 3B ).

**Fig. 4:**
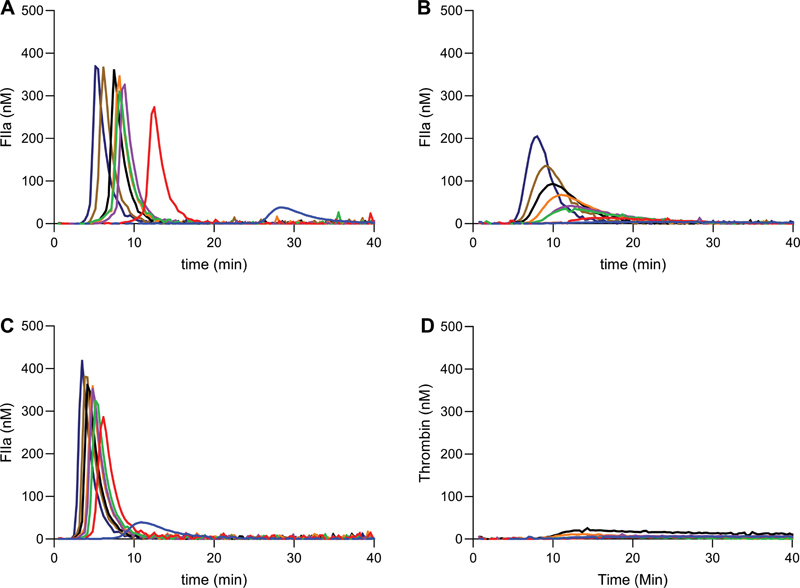
Factor FXIa titration in pooled severe hemophilia A plasma and normal pooled plasma. Thrombin generation in normal pooled plasma and severe hemophilia A plasma was measured. ( **A** ) Normal plasma was titrated with FXIa. ( **B** ) Severe hemophilia A plasma was titrated with FXIa in presence of 1 pM of TF. ( **C** ) Normal plasma was titrated with FXIa in presence of 1 pM of TF. FXIa concentrations: 0 pM (blue), 25 pM (red), 50 pM (green), 75 pM (purple), 100 pM (orange), 150 pM (black), 250 pM (brown), and 500 pM (navy blue). ( **D** ) Severe hemophilia A plasma was titrated with FIXa in presence of 1 pM of TF. FIXa concentration (panel D): 0 pM (blue), 50 pM (red), 100 pM (green), 250 pM (purple), 500 pM (orange), and 1 nM (black). Averages of duplicate experiments are shown. FXIa, activated factor XI; TF, tissue factor.

**Fig. 5:**
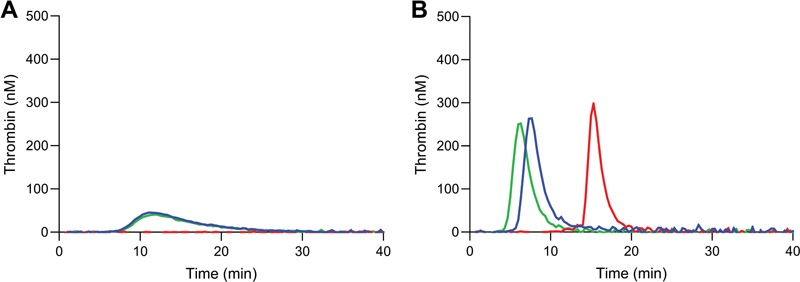
Thrombin generation in pooled severe hemophilia A plasma activated by FXIa in combination with either TF, FXa, or thrombin. For each trigger combination, thrombin generation was measured in presence ( **A** ) and absence ( **B** ) of 5% added FVIII. Triggers: 100 pM FXIa/1 pM TF (blue); 100 pM FXIa/5 pM thrombin (red); 100 pM FXIa/12.5 pM FXa (green). Averages of duplicate experiments are shown. FXIa, activated factor XI; TF, tissue factor.

To increase sensitivity of TGA for lower levels (<10%) of FVIII, alternative conditions for initiation of coagulation were investigated. Activation of TGA by addition of only FXIa was measured in both NP plasma and severe hemophilia plasma. NP plasma showed a dose-dependent increase in TG; however, addition of FXIa to severe hemophilia plasma did not result in measurable TGA curves (data not shown). Only in presence of low amounts of TF (1 pM), FXIa-dependent TG was observed ( Fig. 4A, B ). This observation was most likely explained by activation of low FVIII levels by thrombin generated through extrinsic activation. In contrast to NP plasma, higher concentrations of FXIa were needed to generate baseline TG curves in pooled HA plasma ( Fig. 4B, C ). Based on the results, 100 pM of FXIa, in combination with 1 pM TF, was chosen as the optimal condition, as reliable TG was obtained with room for upward and downward modulation. We next assessed possible replacement of FXIa with FIXa. In a similar setup as with TF/FXIa, TF/FIXa was unable to generate sufficient TG in severe HA plasma ( Fig. 4D ). Alternatively, the replacement of the accessory component TF was investigated by exchanging it for either FXa or thrombin ( Fig. 5 ). TF/FXIa and Xa/FXIa showed TG in severe HA plasma, which was enhanced by the addition of 5% FVIII in a comparable way. In contrast, thrombin/FXIa showed comparable results to TF/FXIa and Xa/FXIa in the presence of 5% FVIII, but not in absence ( Fig. 5 ). In order to obtain the most sensitive TG conditions, TF was chosen over FXa.

**Fig. 6:**
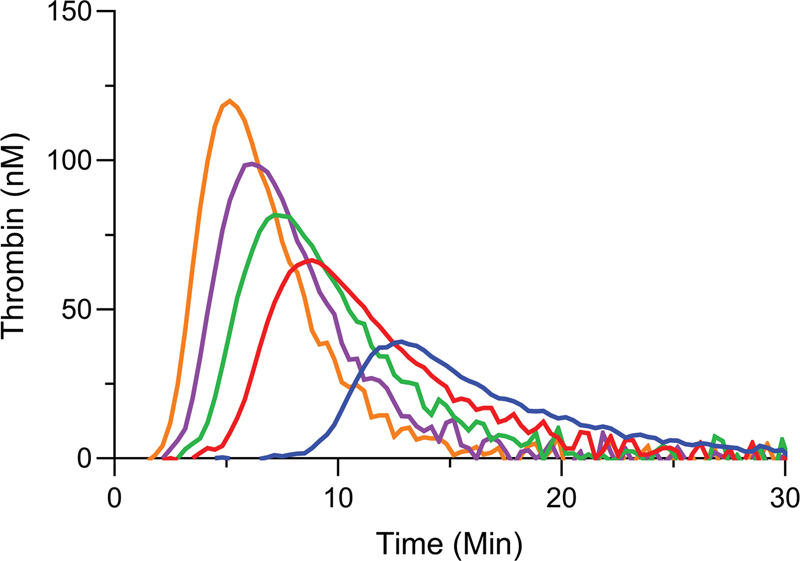
Tissue factor titration in severe hemophilia plasma in presence of FXIa. Thrombin generation in pooled severe hemophilia A plasma was initiated by addition of 100 pM FXIa and various concentrations of tissue factor. Tissue factor was added in concentrations of 1 pM (blue), 2 pM (red), 3 pM (green), 4 pM (purple), and 5 pM (orange). Averages of duplicate experiments are shown. FXIa, activated factor XI.

In order to assess the optimal concentration of TF, a titration was performed in the presence of the chosen fixed FXIa concentration (100 pM) in severe HA plasma ( Fig. 6 ). As only an increase in TG is expected as a result of higher FVIII levels, the lowest amount of TF (1 pM TF) was chosen for further experiments.

To investigate the effect of inhibition of contact activation on the TF/FXIa dual activated TGA, FVIII titration in the TF/FXIa-activated (1 pM/100 pM) TGA was performed in presence and absence of a contact activation inhibitor ( Supplementary Fig. S1 , available in the online version). No obvious differences were seen between absence and presence of contact activation inhibitor, in contrast to results shown in absence of FXIa ( Fig. 1 ). The higher baseline TG in absence of added FVIII is explained by traces of FVIII replacement therapy resulting in FVIII of 2% rather than ideal trough levels of <1%. Although there were no differences in TG as a result of TICA addition in TGA measurements in this experiment, differences in contact activation between several blood withdrawals from individual patient with variable work-up time might still lead to variable levels of contact activation and resulting FXIa levels. Thus, it was decided to maintain TICA in the blood-drawing tubes.

**Fig. 7:**
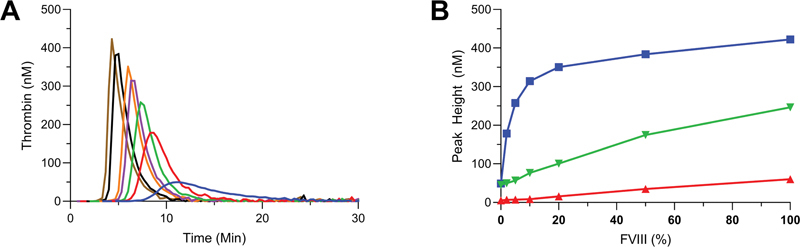
FVIII titration in TF/FXIa-activated thrombin generation. Thrombin generation in pooled severe hemophilia plasma was initiated by 100 pM of FXIa and 1 pM of TF. Addition of FVIII was performed in increments of 0% (blue), 2% (red), 5% (green), 10% (purple), 20% (orange), 50% (black), and 100% (brown), respectively ( **A** ). Averages of duplicates are shown. Panel ( **B** ) shows FVIII sensitivity shown as peak height for 1 pM TF (red), 3 pM TF (green), and 100 pM FXIa/1 pM TF (blue)-activated thrombin generation. Averages of duplicate experiments are shown. FXIa, activated factor XI; TF, tissue factor.

Using the described method with the addition of both FXIa and low TF initiation (the dual-activated TGA), we performed a FVIII titration in our pooled hemophilia plasma ( Fig. 7A ). Compared to the TF-only TGA setup ( Fig. 3 ), the sensitivity for FVIII was significantly increased. The increase in sensitivity to FVIII especially applied to lower, clinically relevant FVIII levels (<10%). Within the titration range, both peak height and ETP increased in a dose-dependent manner, while lag times decreased with increasing amounts of FVIII. To further explore the benefit for dual TGA activation, current conditions were compared to FVIII titrations in absence of FXIa ( Fig. 7B ). In the presence of 1 and 3 pM TF and in the absence of FXIa, the sensitivity for FVIIIa in the lower range (<10%) was almost completely lost ( Fig. 7B ). The decrease in both peak height and ETP in absence of exogenous FXIa emphasized the critical priming role of FXIa in this assay.

To investigate application of the assay to individual patients rather than pooled plasma, FVIII titrations in individual patients were performed ( Supplementary Fig. S2 , available in the online version). The 10 individual severe hemophilia patients that constitute the HAPP with trough levels between <1 and 3% were measured individually as a function of added FVIII. All 10 measurements of patient's plasma showed reliable baseline curves. Subsequent FVIII titration showed an increase in TGA in a similar pattern compared to the average pool measurements. In all individual patient samples, TGA showed similar sensitivity to the addition of FVIII in the lower ranges. All curves leveled off around 20% FVIII after which a gradual shallow, more linear increase in TG was seen up to the final spiked concentration of 100% FVIII.

## Discussion

Measurement of TG in HA plasma can be a challenging effort. Due to low amounts of FVIII present in HA plasma, it is tempting to overstimulate plasma samples with high concentrations of TF to obtain measurable and reproducible TG curves. However, overstimulation with TF will irrevocably lead to direct TG through extrinsic prothrombinase and thereby reduce the sensitivity for the intrinsic pathway. When in turn TF concentrations are reduced in severe hemophilia plasma, TGA curves will become indiscriminate or, in some situations, even will become absent. 10 11 One could argue that these issues might be only relevant in a small subset of the hemophilia patient samples, mostly at trough level situations of severe hemophilia patients. However, this is exactly the population where improved sensitivity of coagulation measurements is needed to aid clinical decision making and could provide the most clinically relevant benefit.

The goal of this study was to optimize the use of TGA in the HA population by increasing the sensitivity for the impaired intrinsic pathway. The optimized TGA should be able to assess coagulation potential in the lower FVIII levels, mimicking trough levels in patients. For reference purposes, a pool plasma consisting of 10 different severe HA patients was created. The hemophilia pool plasma offered a better reference for hemophilia patients as a group rather than individual patients.

During the setup of the assay, we specifically looked at the quality of the resulting TGA curves in each of the development stages. We consider the shape of TGA curves to be essential for correct data interpretation, although this is a vastly overlooked aspect of the assay. 12 Due to low amounts of thrombin formed in HA plasma, especially when using regular low TF-only activated TGA, a proper coagulation pattern consisting of initiation, propagation, and inhibition might not occur, which is reflected in an aberrant curve shape. However, the Thrombinoscope will still provide its calculated parameters like peak height and ETP. To better understand the quality of the data, it is essential to either show or describe the quality of curves in TGA for diagnostic research, especially in hypocoagulant states such as hemophilia.

The benefit of contact activation inhibitors in standardizing the assay was subsequently investigated. Standardizing and controlling analytical and preanalytical variables in TGA have been shown to drastically improve assay variability. 6 13 One of the most significant and uncontrolled variables in TGA, especially in hemophilia, is contact activation. As such, the use of contact activation inhibitors has already been recommended for TGA within the HA patient population. 8 The extent of contact activation can vary greatly. It is influenced by a multitude of factors including blood withdrawal, transport of samples, sample handling (time), and materials used. The level of contact activation directly impacts the amount of intrinsic activation of the sample. Our data showed that TGA is significantly influenced by the amount of contact activation, especially in the currently used TF-only assay. Intrinsic activation of the coagulation pathway increases sensitivity for FVIII, as does contact activation. However, the amount of contact activation per sample is variable and difficult to quantify. Addition of a contact activation inhibitor is therefore crucial in controlling the amount of intrinsic activation and thereby increasing reproducibility. In addition, adding an amount of 100 pM FXIa as an intrinsic activator leads to controlled and reproducible TG in the plasma of hemophilia patients.

The focus on TGA initiation through the intrinsic pathway was chosen to make use of levels of FVIII as the rate-limiting step of HA plasma to provide a new functional HA assay within its specific plasma environment. This is especially relevant in the context of new and emerging treatments for HA, which are mostly non-FVIII therapies. Interestingly, both TF/FXIa- and FXa/FXIa-initiated TG gave similar TG curves, strengthening the hypothesis that both intrinsic and extrinsic activators are required for increased assay sensitivity in the lower FVIII ranges. Increasing the sensitivity for the intrinsic pathway in the optimized dual TF/FXIa-activated TGA in HA has several advantages. The most clinically relevant changes in coagulation potential are seen in severe hemophilia patients. Small increases of coagulation potential, for example by medication, can drastically improve the bleeding phenotype, whereas a similar increase in coagulation potential in mild hemophilia patients will most often not modify the bleeding phenotype at all. With the current TGA setup, we are now able to better measure TGA on these clinically FVIII ranges.

Although TF-only activated TGA in severe HA plasma does not seem to possess the required sensitivity to show small changes in coagulation potential, it might still be useful in some settings. Several studies showed its use in patient monitoring, 3 4 14 15 16 17 bypassing agent dosing 18 19 20 21 22 and evaluation of novel treatments. 23 24 25 26 27 Due to the low sensitivity of TF-activated TG for low amounts of FVIII, currently only studies are performed that either measure large changes in coagulation potential by adding larger amounts of FVIII or measure averages in large population-based studies. Implementing the optimized dual TF/FXIa-activated TGA will aid current and future uses of TG in predicting clinical efficacy of treatment in the individual HA patient by providing a baseline measurement and additional sensitivity for small increases in coagulation potential by factor replacement or alternative treatment strategies even at low FVIII ranges.

## Conclusion

We propose optimization of the TGA in severe HA by dual-activation of the assay by TF/FXIa leading to increased sensitivity toward added FVIII compared to activation by TF only, especially in lower FVIII ranges. The proposed setup allows for more sensitive and reproducible measurements at low, clinically relevant, FVIII levels than the currently used TF-only activated assay. With many new therapeutic options for hemophilia being introduced recently, it has become increasingly important to have a robust and sensitive assay to measure the coagulation potential of HA patients. The increased sensitivity of the optimized dual TF/FXIa-activated TGA might allow for better individual characterization at baseline, interventions, and follow-up regardless of treatment strategies.

## Notes

**What is known about this topic?**

- Adequate assessment of thrombin generation at trough levels in severe hemophiliacs is hampered by current protocols.
- Thrombin generation is able to differentiate between plasmas from severe, moderate, and mild hemophiliacs.
- Contact activation inhibition is a relevant determinant of thrombin generation in severe hemophilia A.

**What does this paper add?**

- Optimized protocols for thrombin generation allow for measurement of trough levels.
- Dual TF/FXIa activation of thrombin generation enables high sensitivity assessment of plasma from individuals within severe hemophilia A populations.
- The variable unknown amount of contact activation in plasma can be blocked by contact activation inhibitors, while maintaining a controlled amount of intrinsic activation by addition of FXIa.
