# Supplementary material for: Sensitive Measurement of Clinically Relevant Factor VIII Levels in Thrombin Generation Assays Requires Presence of Factor XIa
Source: Thromb Haemost. 2023 Jul 10;123(11):1034–41. doi: 10.1055/a-2101-7961 (PMC10615588; doi:10.1055/a-2101-7961)
Supplement: Supplementary file 1 — Supplementary Material [file 10-1055-a-2101-7961-s22060326.pdf]

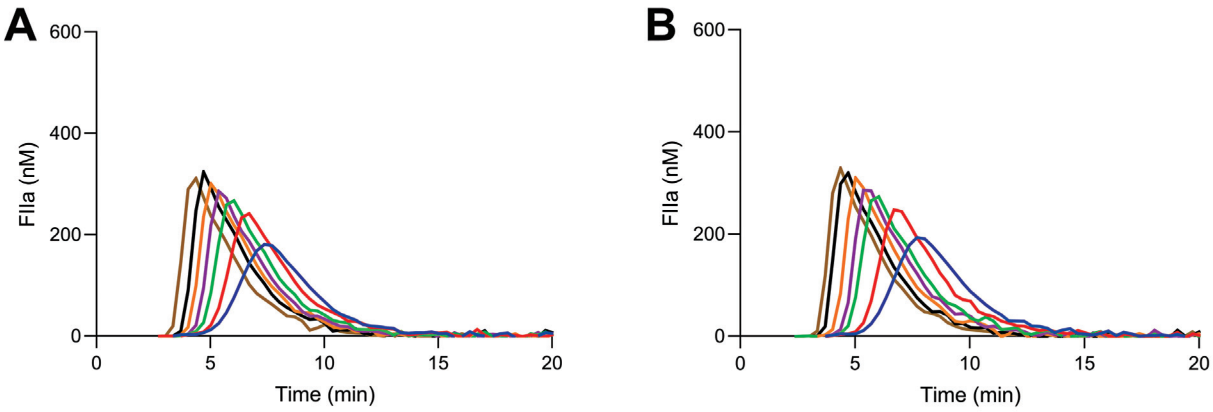

Supplementary Fig. S1 Effect of contact activation on TF/FXIa-activated thrombin generation in severe hemophilia A plasma. Thrombin generation assay was performed in TF/FXIa-activated single individual severe hemophilia plasma in absence (**A**) and presence (**B**) of contact activation inhibitor. The plasma was spiked with 0% (blue), 2% (red), 5% (green), 10% (purple), 20% (orange), 50% (black), or 100% (brown). Averages of duplicate experiments are shown. FXIa, activated factor XI; TF, tissue factor.

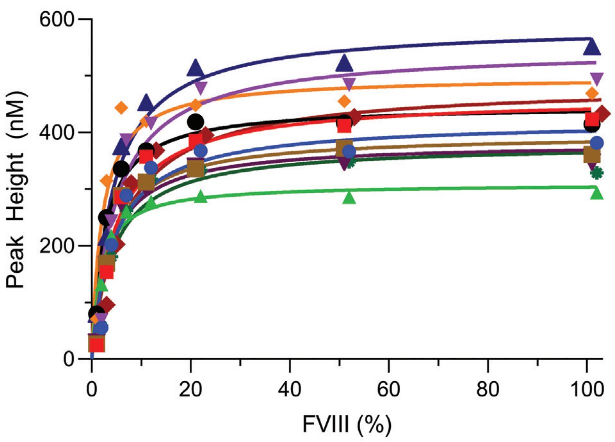

Supplementary Fig. S2 Baseline coagulation potential and FVIII titration in severe hemophilia A patients can be measured using TF/FXIa-activated thrombin generation. Thrombin generation in pooled severe hemophilia plasma was initiated by 100 pM of FXIa and 1 pM of TF. In vitro spiking of the plasma with FVIII was performed in increments of 0, 2, 5, 10, 20, 50, and 100%, respectively. Measurements were performed in our pooled severe hemophilia A plasma and the 10 individual severe hemophilia A patients of which the pooled consisted. Measured peak heights are shown as individual dots, additionally a fitted curve has been added for each of the measurements. The pooled plasma is shown in blue. The patients are shown in alternative colors. Averages of duplicate experiments are shown. FXIa, activated factor XI; TF, tissue factor.

 © 2023. The Author(s).
